# Simple and Cost-Effective Design of a THz-Metamaterial-Based Hybrid Sensor on a Single Substrate

**DOI:** 10.3390/s25123660

**Published:** 2025-06-11

**Authors:** Uddipan Nath, Sagnik Banerjee, Carlo Santini, Rocco Citroni, Fabio Mangini, Fabrizio Frezza

**Affiliations:** 1 ICT and Internet Engineering, Department of Electronics Engineering, University of Rome “Tor Vergata”, 00133 Rome, Italy; uddipan.nath@students.uniroma2.eu; 2 Department of Information Engineering, Electronics and Telecommunications (DIET), Sapienza University of Rome, 00184 Rome, Italy; banerjee.2055414@studenti.uniroma1.it (S.B.); carlo.santini@uniroma1.it (C.S.); rocco.citroni@uniroma1.it (R.C.); 3 Department of Engineering, Niccolò Cusano University, 00166 Rome, Italy; fabio.mangini@unicusano.it

**Keywords:** metamaterial, temperature sensor, refractive index sensor, absorber

## Abstract

This study presents a cost-effective Hybrid Metamaterial Absorber (HMA) featuring a simple circular-patterned cylindrical design, comprising an indium antimonide (InSb) resonator on a thin copper sheet. Through numerical simulations, we demonstrate that the structure exhibits temperature-tunable properties and refractive index sensitivity. At 300 K (refractive index = 1), a peak absorption of 99.94% is achieved at 1.797 THz. Efficient operation is observed across a 40 K temperature range and a refractive index spectrum of 1.00–1.05, relevant for thermal imaging and spatial bio-sensing. The simulated temperature sensing sensitivity is 13.07 GHz/K, and the refractive index sensitivity is 1146 GHz/RIU. Parametric analyses reveal tunable absorption through adjustments of the InSb resonator design parameters. Owing to its high efficiency and sensitivity demonstrated in simulations, this HMA shows promise for sensing applications in biotechnology, semiconductor fabrication, and energy harvesting.

## 1. Introduction

Academic and industrial research has increasingly focused on terahertz technology in recent years, driven by its promising applications in multiple fields such as wireless communication, biomedical applications, imaging, security scanning, and other cutting-edge domains [1,2,3,4,5]. However, the limited number of natural materials that respond to THz vibrations presents challenges for advancing this technology. Engineered periodic architectures, termed metamaterials, are specifically designed to exhibit extraordinary electromagnetic properties including artificial magnetism, non-specular reflection phenomena, and negative index refraction. Its capacity to absorb incoming electromagnetic radiation is one such characteristic. These structures, known as electromagnetic absorbers, have drawn interest from academics due to their wide range of uses across several frequency bands [6]. The unit cells, suitably replicated in periodic patterns in two or three dimensions, comprise a Terahertz Metamaterial Absorber (TMA). Each unit cell can be composed of multiple layers of metal and dielectric materials, often ranging from two to three or more. However, their intricate geometries and high fabrication costs may restrict their practicality, especially in sensing applications. Numerous research teams have worked to improve the absorption capacity of practically realizable Metamaterial Absorbers (MMA) used in THz sensing applications [7,8,9,10,11,12,13,14,15,16,17,18,19,20,21,22].

THz Quantum Cascade Lasers (QCLs) are unipolar semiconductor lasers capable of emitting coherent, tunable radiation in the THz band through inter-subband transitions in quantum well heterostructures [23,24]. They provide narrow linewidths (often <10 MHz), high output power (tens of mW), and electrical tunability—features that make them highly desirable for compact, high-resolution spectroscopic platforms [25]. In addition, advances in dispersion engineering have enabled the development of THz frequency combs based on QCLs, providing phase-locked emission across a broad range of frequencies [26,27]. These combs offer ultrafast acquisition rates, absolute frequency referencing, and multiplexed spectral coverage, which are essential for resolving complex biomolecular fingerprints [28].

This integration offers several compelling advantages for biosensing: Spectral alignment between QCL frequency and metamaterial resonance allows maximal excitation of localized plasmonic or dielectric modes, leading to enhanced biomolecular sensitivity. Monolithic or quasi-monolithic integration reduces alignment complexity and supports chip-scale platforms suitable for portable diagnostics. High SNR and fast acquisition from coherent QCL emission enable real-time detection with sub-picogram limits of detection. Tunable or comb-like excitation allows multiplexed probing of multiple biomolecular absorption lines or interactions simultaneously.

TMAs are commonly utilized to measure the refractive index of the surrounding environment. For example, a biosensor featuring a square ring resonator (SRR) with a refractive index sensitivity of 300 GHz/RIU was proposed in [20], while a TMA-based biosensor incorporating four undifferentiated resonators in a single unit cell demonstrated a sensitivity of 85 GHz/RIU [21]. However, these TMAs exhibit relatively low sensitivity compared to our HMA and are limited to refractive index sensing, lacking the capability for temperature sensing. On the other hand, TMAs have also been engineered to function as temperature sensors by responding to thermal variations. Notable developments in this field comprise a 3D perfect metamaterial absorber by Zou et al. exhibiting a 15.24 GHz/K temperature sensitivity [9], alongside Luo et al.’s work on bilayer all-dielectric InSb star resonator absorbers achieving a 9.6 GHz/K sensitivity [17]. Despite their performance, these TMAs suffer from complex designs that are challenging to fabricate, and the use of gold as the metallic component makes them less cost-effective compared to our HMA. Additionally, they are restricted to temperature sensing and cannot operate as refractive index sensors.

Similarly, to our HMA, other hybrid metamaterial absorbers have been proposed. For example, F. Chen et al. designed a hybrid narrow-band metamaterial sensor capable of dual functionality as both a refractive index and temperature sensor, with sensitivities of 960 GHz/RIU and 2.13 GHz/K, respectively [14]. P. Agarwal et al. presented a dual-band metasurface based on an InSb micro-rod, achieving refractive index sensitivities of 1900 GHz/RIU and 1800 GHz/RIU, alongside temperature sensitivities of 5.5 GHz/K and 7.5 GHz/K [15]. Additionally, Y. Cheng et al. developed a narrow-band HMA sensor using all-dielectric InSb, with refractive index and temperature sensitivities of 920 GHz/RIU and 4.2 GHz/K, respectively [16]. While these HMAs demonstrate respectable refractive index sensitivity, their temperature sensitivity is significantly lower compared to our proposed HMA.

In contrast to previous studies, this work introduces a novel HMA comprising an InSb resonator integrated with a thin copper sheet. This hybrid metamaterial design achieves dual operational modes, simultaneously permitting thermal tuning and precise monitoring of environmental refractive index variations. A key advantage of this design lies in its simplicity, featuring a substrate patterned with cylindrical structures mounted on a copper sheet, which ensures cost-effectiveness. Numerical simulations reveal that the absorber achieves an absorption rate of 99.94% at 1.797 THz under conditions of 300 K external temperature and a refractive index of 1. The HMA demonstrates efficient operation across a broad temperature range of 40 K and a refractive index range of 1.00 to 1.05, maintaining a high absorption rate. This refractive index range is particularly significant, as it encompasses the detection of various harmful gases [29]. As a temperature sensor, the HMA achieves a sensitivity of 13.07 GHz/K, while as a refractive index sensor it delivers an exceptional sensitivity of 1146 GHz/RIU. The sensor demonstrates exceptional performance as both a temperature and refractive index sensor, characterized by high Q-factor, absorption efficiency, and sensitivity. Its ability to operate across a range of refractive indices makes it highly promising for thermal imaging [30] and spatial bio-sensing [16]. Additionally, its temperature sensing capability enables its use in industrial process control, such as monitoring thermal uniformity in semiconductor manufacturing, and in security screening for detecting concealed objects like explosives through temperature anomalies.

The article is structured as follows: in Section 2, we outline the unit cell’s construction and geometrical features as well as the setup of the numerical simulation program used to analyze its electromagnetic properties. Section 3 summarizes the results of simulations on the absorption capacities and resonance of the suggested structure, which support our findings on the metamaterial characteristics of our design. This paper’s results are finally presented in Section 4, where we also discuss the study’s potential applications and further advances.

## 2. Materials and Methods

**Figure 1:**
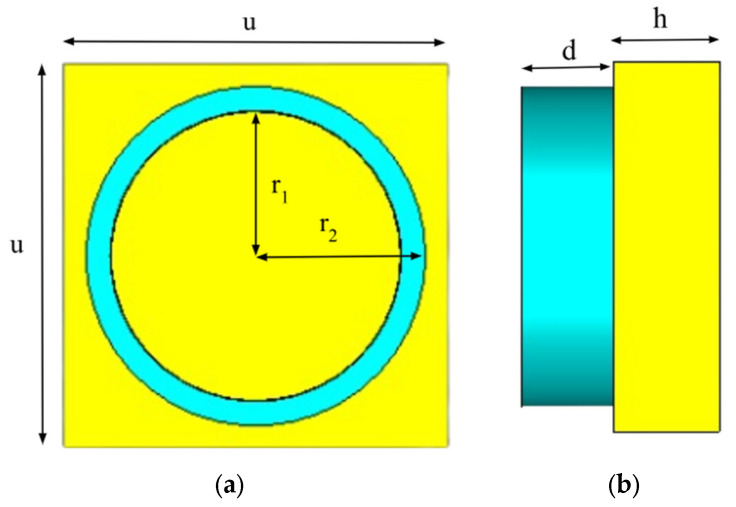
Design of proposed TMA: (a) view from top; (b) view from side.

The configuration of the proposed HMA unit cell is described in Figure 1a,b. The front and side views of the structure are illustrated in Figure 1a and Figure 1b, respectively. The proposed HMA comprises a two-layered structure. The top layer resonator is made up of Indium antimonide (InSb) semiconductor material with a thickness of d = 20 μm. The bottom layer consists of a copper (annealed) metal sheet having conductivity σ = 5.8 × 10^7^ [S/m] and thickness h = 23 μm. The solid metal sheet at the bottom prevents electromagnetic radiation from passing through. In Figure 1a, the InSb cylindrical structure is depicted, with an inner radius of r_1_ = 30 μm and an outer radius of r_2_ = 35 μm. The square-shaped unit cell has a dimension of u = 80 μm. Table 1 shows the different design parameters used and their corresponding magnitudes in μm.

InSb is a well-known thermo-sensitive semiconductor material, with its permittivity (ε(ω)) and intrinsic carrier density (N) being influenced by changes in the external environmental temperature [15]. This unique property makes InSb particularly suitable in the design of thermally tunable metamaterial absorbers operating within the terahertz frequency range. The temperature-dependent and dispersive behavior of InSb’s complex permittivity is accurately characterized by the Drude model, which is given by Equations (1)–(3) [2,5,6,7].

(1) $$\varepsilon \left(\omega \right)=\varepsilon_{\infty }-\frac{\omega_{p}^{2}}{\omega^{2}+i\gamma \omega }$$

(2) $$\omega_{p}=\sqrt{\frac{Ne^{2}}{0.015\;m_{e\;}\varepsilon_{0}}}$$

(3) $$\gamma =\frac{e}{0.015\;m_{e}\mu }$$

(4) $$N=5.76\times 10^{20}T^{3/2}e^{\left(-0.13/k_{B}T\right)}$$

**Figure 2:**
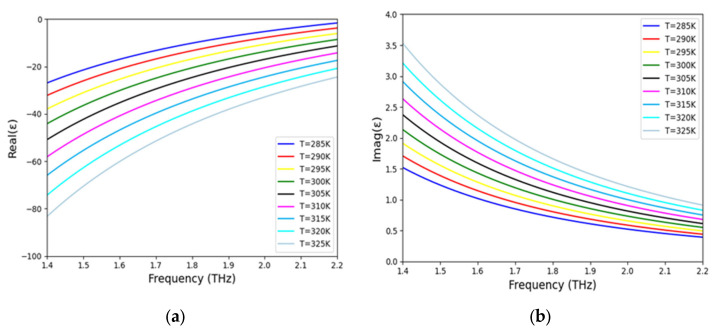
Temperature-dependent analysis of the relative permittivity of InSb is presented for (**a**) the real and (**b**) the imaginary parts.

In Equations (1)–(4), ω is the angular operating frequency, ε_ꝏ_ is the high frequency bulk permittivity whose value for InSb results to be ε_ꝏ_ = 15.68, ω_p_ is the plasmonic frequency (defined by Equation (2)), γ is the damping constant (defined by Equation (3)), *e* is the electron charge, m_e_ is the electron mass, ε_0_ is the free-space permittivity, μ is the electron mobility, *T* is the temperature in Kelvin, and k_B_ is the Boltzmann constant. The intrinsic carrier density of InSb, N, is defined by Equation (4), it describes the temperature-dependent carrier density N, where the exponential term e^−0.13/(k^_B_^T)^ dominates the thermal sensitivity. This reflects the rapid increase in carrier concentration with rising temperature due to thermal excitation. It has been observed that electron mobility shows slight variation with temperature between 160 K and 360 K. Consequently, the value of γ is considered constant at 0.1π THz [17,19]. When the temperature is between 160 and 350 K and the frequency is between 0.1 and 2.2 THz, these equations can be used [6,7,8]. Figure 2a,b present the calculated real and imaginary components of ε(ω) for InSb over the working frequency range of 1.4 to 2.2 THz, demonstrating high sensitivity to temperature (T). It has been observed that the values of Re(ε) and Im(ε) gradually increase and decrease, respectively, as the working frequency increases from 1.4 to 2.2 THz. Conversely, the magnitude of Re(ε) decreases, and Im(ε) increases with a rise in temperature.

## 3. Results and Discussions

### 3.1. Absorption Theory and Analysis

The absorber structure was designed and simulated in CST Microwave Studio 2024 using periodic boundary conditions to model an infinite array of unit cells [18]. When an electromagnetic wave interacts with the absorber surface, the principle of energy conservation requires that the incident power be distributed among reflected, transmitted, and absorbed components, as expressed by the fundamental relationship

(5) $$R\left(\omega \right)+T\left(\omega \right)+A\left(\omega \right)=1$$

**Figure 3:**
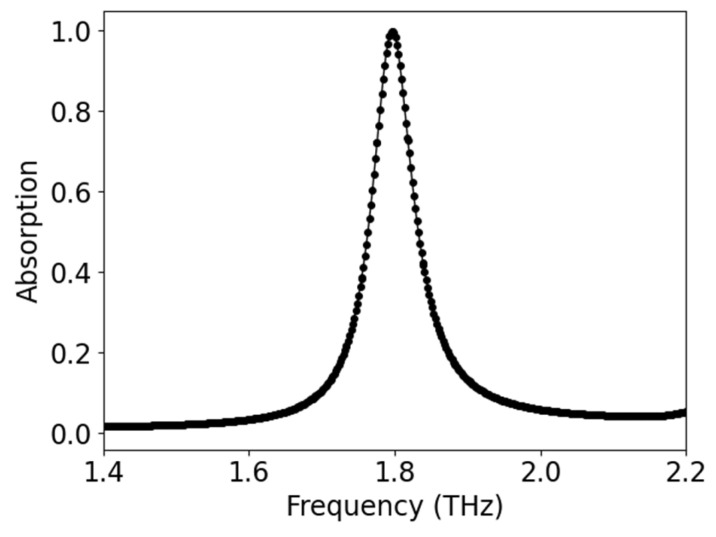
Peak absorption at resonance for the proposed metamaterial absorber at 300 K.

Here, *A(ω)* represents absorption, while *R(ω) = |S_11_(ω)|^2^* and *T(ω) = |S_21_(ω)|^2^* quantify the reflected and transmitted power, respectively. This equation highlights the critical design parameters for achieving optimal absorption performance, where maximum absorption (A(ω) ≈ 1) occurs when both reflection and transmission coefficients are simultaneously minimized [7,10]. The careful balance of these components forms the theoretical foundation for evaluating the absorber’s efficiency and guides the interpretation of subsequent simulation results. Figure 3 reports the simulated peak of absorption for the proposed metamaterial absorber at the resonance frequency (1.797 THz) at 300 K, which gives 99.94% absorption.

**Figure 4:**
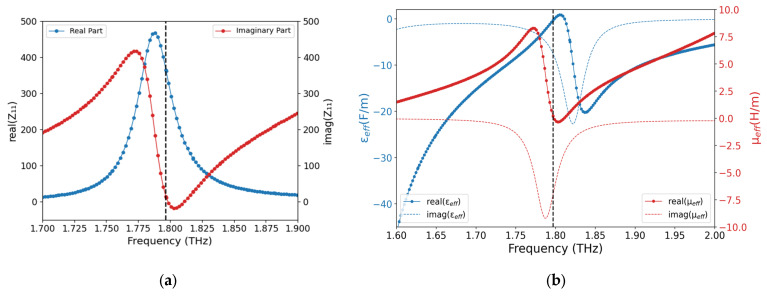
(**a**) Plot of surface impedance as exhibited by the absorber at 300 K; (**b**) effective medium parameters for the designed absorber at 300 K.

Figure 4a shows the plot of effective impedance as a function of frequency. At resonance frequency, the impedance is given by Z = R + jX = 361.62 + j12.07 Ω, which clearly indicates that, at resonance, the resistive part, R, approaches free-space impedance of 377 Ω. The reactive part, X, is positive and close to zero at resonance and becomes slightly negative a little beyond resonance frequency, corresponding to a partial capacitive behavior, which becomes highly inductive. The impedance matching condition is almost fulfilled at resonance, since the absolute value of impedance becomes 361.83 Ω.

The proposed device does not suffer from back-transmission due to the higher skin depth of copper compared to the wavelength of the incident radiation. Since the structure completely blocks transmission *(T(ω) = |S_21_(ω)|^2^ = 0)*, Equation (5) reduces to the simplified form presented in Equation (6).

(6) $$R\left(\omega \right)+A\left(\omega \right)=1\;or\;A\left(\omega \right)=1-R\left(\omega \right)\;or\;A\left(\omega \right)=1-{\left|S_{11}\left(\omega \right)\right|}^{2}$$

Since reflection is minimized due to impedance matching, near-perfect absorption is achieved at the resonant frequency.

Figure 4b reports the behavior of the effective medium parameters as a function of frequency at 300 K. At resonance, the real part of the permittivity is negative while the permeability has a negligible impact, and, hence, the proposed absorber shows an epsilon-negative (ENG) metamaterial nature. The absorber behaves as an electric plasma where the resonance is brought about by the quantized plasmons.

### 3.2. Effect of the Material and Structure Characteristics on Perfect Absorption

**Figure 5:**
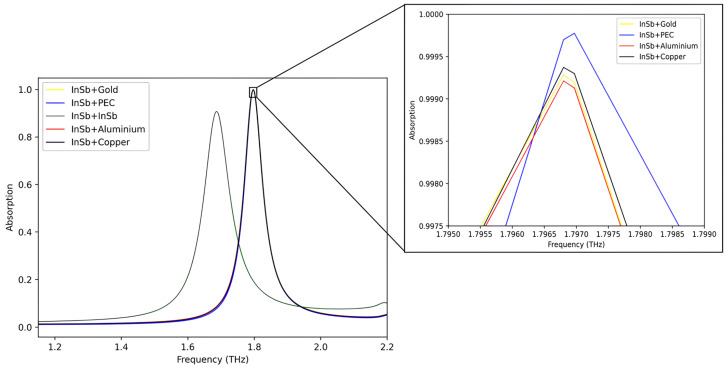
A comparison of the absorption spectra for various combinations of materials.

In Figure 5, the absorption spectra of five different material combinations at a temperature of 300 K and at unity refractive index corresponding to free-space are presented to demonstrate the contributions of each material towards complete absorption. Absorbers incorporating InSb resonators with copper (InSb + Copper), aluminum (InSb + Aluminum), gold (InSb + Gold), or perfect electric conductor (InSb + PEC) metal sheets exhibit nearly identical absorption responses, suggesting minimal contribution of conductive power loss to peak absorptivity. However, a closer analysis of the absorption peaks reveals that the InSb + Copper combination achieves the highest absorptivity. In contrast, the structure made entirely of InSb (InSb + InSb) shows the lowest absorptivity, reaching only 90.75%. These results confirm that the inherent dielectric losses of the InSb material significantly influence the absorber’s performance. Notably, the best results are obtained when the InSb resonator is combined with a copper sheet, which also makes this HMA a cost-effective solution.

**Figure 6:**
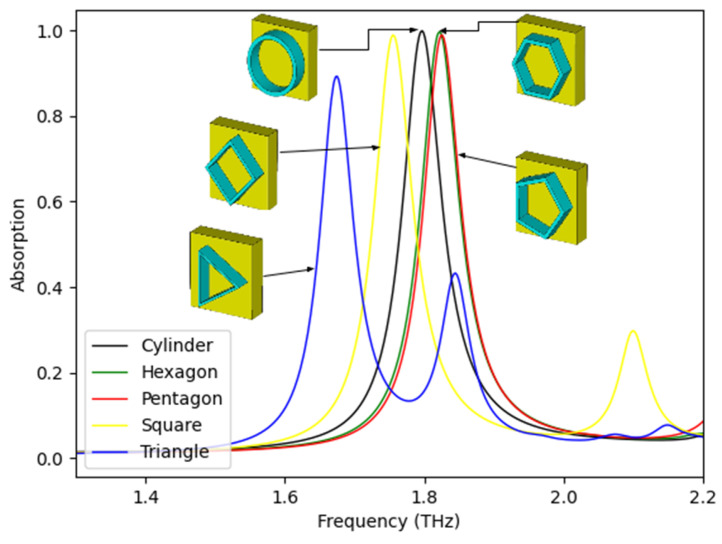
A comparison of the absorption spectra for various combinations of the shape of the substrate of the absorber.

To gain a deeper understanding of the absorption spectrum, simulations were conducted to analyze the resulting absorptivity for different shapes of the InSb resonator. Results are reported in Figure 6, where the absorption spectrum for five different resonator structures is shown. Different resonator shapes—such as a round cylinder or prisms with triangular, square, pentagonal, or hexagonal cross sections displayed nearly identical absorption spectra, suggesting that energy dissipation due to conduction is negligible at peak absorption. However, a closer examination of the absorption peaks reveals that the cylindrical resonator with round cross section (cylinder) exhibits the highest absorptivity, while the structure featuring a triangular cross section demonstrates the lowest absorptivity, achieving only 89.30%. These findings clearly demonstrate that the HMA has an optimal and simple absorber structure.

### 3.3. Parametric Analysis

**Figure 7:**
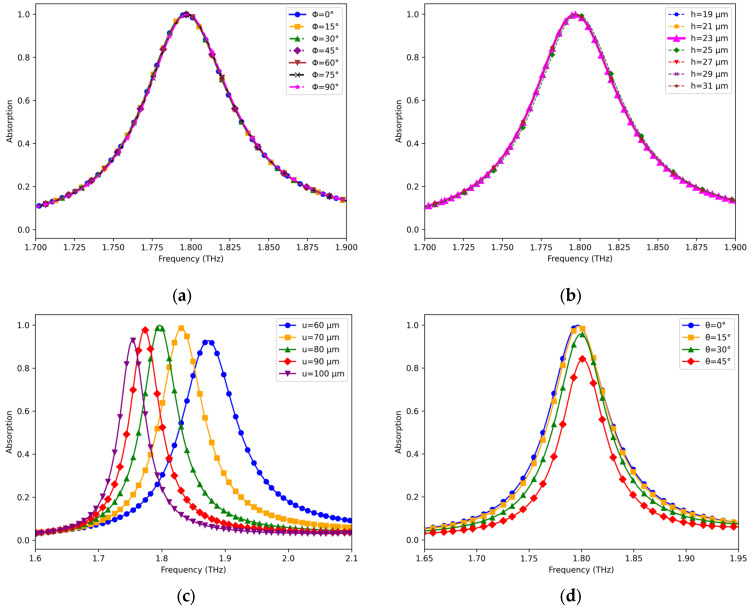
Analyses of used parameters: (**a**) polarization angle; (**b**) height of dielectric spacer; (**c**) periodicity of unit cell; (**d**) incident angle.

The sensor’s practical applicability was evaluated by analyzing its absorption spectrum under varying polarization angles (0–90°), as demonstrated in Figure 7a. The absorption characteristics exhibited remarkable stability, remaining invariant to polarization changes. In all cases, the absorber demonstrated near perfect absorptivity, achieving a value of 99.94%. Absorption levels are slightly dependent on values of the copper film height h as illustrated in Figure 7b. A closest enquiry has shown that the highest absorption level of 99.94% is achieved when the copper film height is set to h = 23 µm. As evidenced in Figure 7c, a unit cell periodicity of u = 80 μm yields optimal performance, achieving both maximum absorption efficiency and superior Q-factor characteristics. A noticeable reduction in absorption occurs when the dimensions are further altered. From Figure 7d, it is observed that the sensing capabilities are enhanced when the incident angle is kept between 0° and 45°. Within this range, the absorption rate remains above 90%.

### 3.4. Surface Current

**Figure 8:**
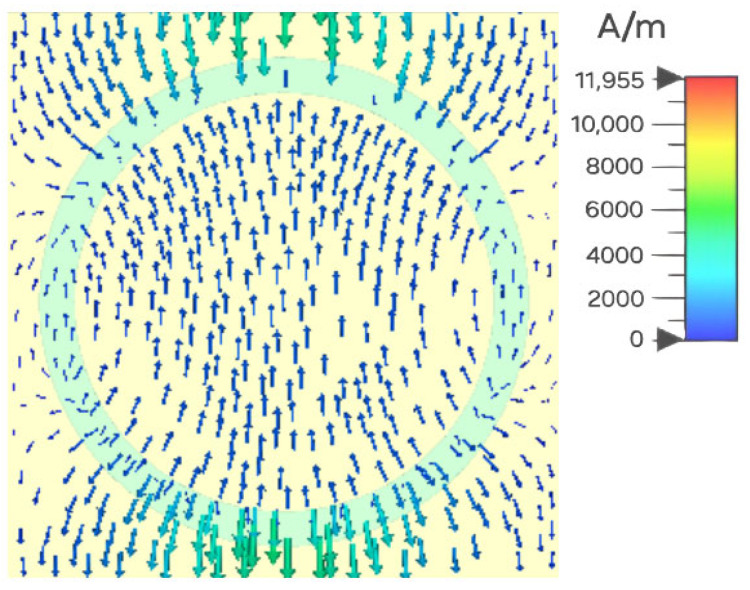
Distribution of surface current for the HMA at f = 1.797 THz and T = 300 K.

To impose periodic boundary conditions, the following considerations apply: When the magnetic field is oriented along the *y*-direction, the *x–z* plane behaves as a perfect magnetic conductor with vanishing tangential magnetic components. If the electric field is polarized along the x direction, the *y–z* plane behaves as a perfect electric conductor, nullifying tangential electric components. This configuration enables plane–wave propagation along the *z*-axis to effectively excite the unit cell. The incoming plane wave in this case generates surface charge, $J_{s}=n\times N$, which causes the magnetic field, N, discontinuous behavior to be at resonance, with n being the outward unit normal vector to the front face of the absorber. The fluctuating or oscillating surface currents resemble a wire antenna current distribution: this explains the resonant nature of the metamaterial. Furthermore, the current density is primarily limited to the upper and lower edges of the InSb resonator layer, as shown in Figure 8. It indicates that, when an incoming THz wave influences the structure at normal incidence, maximum current is induced over the resonator and, hence, the majority of the energy is lost in the resonator in the form of heat because of the inherent dielectric losses of the InSb material, with very little energy reaching the background copper material. The achieved near-perfect absorption at resonance originates from strong air–InSb resonator coupling, enabled through careful impedance matching between the media.

### 3.5. Sensitivity

Table 2 reports peak absorptivity, resonance frequency, FWHM bandwidth, and Q-factor of the suggested HMA at refractive index 1 for different temperatures when the sensor is working as a temperature sensor.

**Figure 9:**
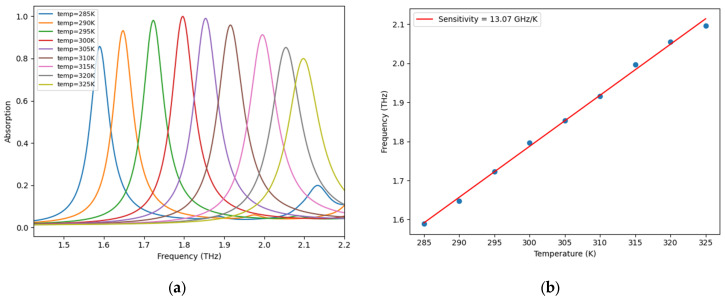
(**a**) Temperature-dependent absorption spectra of the HMA and (**b**) corresponding resonant frequency variation as a function of external temperature.

The temperature sensitivity $S_{T}$, representing the resonant frequency (Δ*f*_0_) shift per degree of temperature variation *(*Δ*T)*, constitutes the primary figure of merit for thermal sensing applications. As derived from Equation (7) and verified through linear regression analysis of the simulated data (Figure 9), the proposed structure achieves a substantial sensitivity of 13.07 GHz/K across the operational temperature range of 285 K to 325 K, evaluated at 5 K increments. This performance demonstrates a consistent positive thermal coefficient, manifesting as a progressive blue-shift in resonant frequency with rising temperature.

(7) $$S_{T}=\frac{\Delta f_{0}}{\Delta T}$$

Moreover, a measurable red-shift of the resonance wavelength is obtained when the refractive index of the encircling medium is varied, that is, as the refractive index increases, the corresponding resonant frequency decreases. Therefore, the proposed metamaterial-based absorber acts as a hybrid sensor capable of detecting temperature and refractive index variation at the same time. The independence from any calibration procedure represents a serious advantage compared to the current state-of-the-art methods. Such behavior is based on well-known fundamental laws of physics stating the inverse relationship between values of the refractive index and the temperature of a medium. Correspondingly, the refractive index sensitivity, $S_{n}$ can be defined as the ratio between the alterations in resonant frequency (Δ*f*_0_) and the alterations in the refractive index (Δ*n*) of the surrounding medium, i.e.,

(8) $$S_{n}=\frac{\Delta f_{0}}{\Delta n}$$

**Figure 10:**
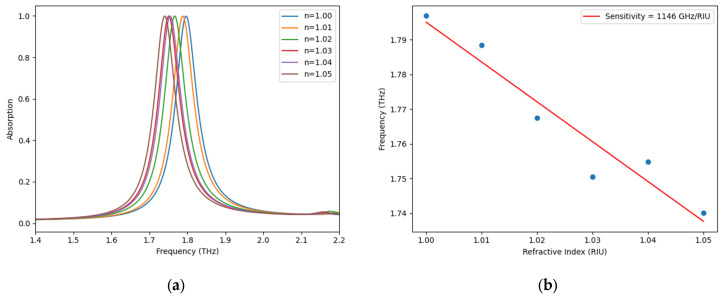
(**a**) Refractive index-dependent absorption characteristics of the HMA and (**b**) corresponding resonant frequency values [THz] versus refractive index.

The quantity in Equation (8), can be evaluated by linear regression applied to simulated data points, as shown in Figure 10. In performed simulations, the refractive index value has been varied in the range from 1.00 to 1.05 within a step width of 0.01: the resulting refractive index sensitivity value resulted to be 1146 GHz/RIU. Although the refractive index range of 1.00 to 1.05 rarely correspond to condensed media, it is highly relevant for spatial bio-sensing, harmful gas detection, and thermal imaging [16,29,30]. Absorption characteristics and resonance frequency values of the suggested HMA as a function of the refractive index of the surrounding medium at 300 K temperature are listed in the following Table 3:

To further advance this research and enhance the sensor’s sensitivity, it is essential to increase its frequency selectivity by achieving narrower absorption peaks with higher quality factors. Several state-of-the-art approaches can facilitate this improvement. For instance, utilizing tunable materials such as vanadium dioxide, whose conductivity can be modulated to produce sharper resonance peaks [31], offers a promising pathway. Another effective strategy involves inducing quasi-bound states in the continuum (quasi-BICs), which are highly localized surface-bound states capable of supporting extremely high-quality factors. Implementing quasi-BICs can significantly elevate sensor sensitivity by enabling more precise resonance detection [32].

Table 4 provides a comparative analysis of the proposed metamaterial absorber against current designs, assessing critical parameters such as sensing performance, material composition, absorption characteristics, thermal and refractive index sensitivity, structural footprint, polarization behavior, and angular tolerance. The presented HMA achieves exceptional performance metrics, featuring near-ideal absorption, remarkable sensitivity to both temperature and refractive index variations, a compact physical profile, an economical two-layer InSb–copper configuration, and a straightforward circular resonator design that delivers consistent high-efficiency operation.

## 4. Conclusions

In this numerical study, we introduce a novel HMA composed of an InSb resonator integrated with a thin copper sheet. The simulated results indicate that the HMA demonstrates bifunctional operation, combining thermally tunable characteristics with environmental refractive index sensing capabilities. A notable advantage of this design is its simplicity, featuring a circular-patterned cylindrical substrate mounted on a copper sheet, ensuring a cost-effective solution. Simulations show that the proposed absorber achieves an absorption rate of 99.94% at 1.797 THz under conditions of 300 K external temperature and a refractive index of 1 of the surrounding media. The HMA operates efficiently across a wide temperature range of 40 K (285–325 K) and a refractive index range of 1.00 to 1.05, maintaining a high absorption rate. The absorption characteristics and Q-factor in response to variations in temperature and refractive index have been assessed through numerical simulations. As a temperature sensor, the HMA achieves a simulated sensitivity of 13.07 GHz/K, while as a refractive index sensor; it delivers an exceptional simulated sensitivity of 1146 GHz/RIU. Additionally, parametric analyses reveal that the absorption profile of the absorber can be precisely controlled by optimizing the design parameters of the InSb resonator. Looking ahead, the proposed THz metamaterial-based hybrid sensor holds promise for diverse applications, including thermal imaging and spatial bio-sensing, environmental monitoring, and industrial process control, owing to its high sensitivity and compact design.

## Figures and Tables

**Table 1:** Parameters used in the proposed structure and its dimensions.

Design Parameter	Magnitude (µm)	Definitions of the Parameters
u	80	Unit cell’s periodicity
h	23	Height of the metal film
d	20	Height of the InSb cylindrical structure
r_1_	30	Inner radius of the InSb cylindrical structure
r_2_	35	Outer radius of the InSb cylindrical structure

**Table 2:** Response of the proposed HMA to temperature changes.

Temperature (K)	Resonance Frequency (THz)	FWHM Bandwidth (THz)	Absorptivity	Q-Factor
285	1.589	0.048	85.70%	33.10
290	1.648	0.056	93.18%	29.43
295	1.723	0.063	98.04%	27.35
300	1.797	0.068	99.94%	26.43
305	1.854	0.072	99.02%	25.75
310	1.917	0.075	95.80%	25.56
315	1.996	0.076	91.23%	26.26
320	2.055	0.075	85.18%	27.40
325	2.097	0.073	79.97%	28.73

**Table 3:** Response of the proposed HMA with the change in refractive index.

Refractive Index	Resonance Frequency (THz)	FWHM Bandwidth (THz)	Absorptivity	Q-Factor
1.00	1.797	0.068	99.94%	26.43
1.01	1.788	0.068	99.95%	26.29
1.02	1.767	0.068	99.96%	25.99
1.03	1.751	0.069	99.98%	25.38
1.04	1.755	0.068	99.99%	25.81
1.05	1.740	0.068	99.97%	25.59

**Table 4:** Comparison of the suggested absorber’s sensing capabilities with those of the current absorbers working at the terahertz frequency range.

References	Temperature Sensor	Refractive Index Sensor	Thickness (µm), Periodicity (µm)	Material Configurations	Absorptivity (%)	Temperature Sensitivity (GHz/K)	Refractive Index Sensitivity (GHz/RIU)	Polarization Sensitive	Incident Angle Stability
[7]	Yes	No	42, 100	InSb–Au	99.94	16.1	N/A	Yes	N/A
[11]	No	Yes	8.4, 102	Au-GaAs-Au	99.0	N/A	1447.0	Yes	N/A
[12]	No	Yes	8.4, 100	Al-GaAs-Al	99.5	N/A	1500.0	No	N/A
[13]	No	Yes	8.7, 80	Al-GaAs-Al	99.8	N/A	187.0	N/A	N/A
[14]	Yes	Yes	139, 150	InSb–Au–Glass	99.9	2.13	960.0	Yes	N/A
[15]	Yes	Yes	160, 150	InSb	98.0, 91.0	5.5, 7.5	1900.0, 1800.0	N/A	N/A
[16]	Yes	Yes	160, 150	InSb	99.9	4.2	920.0	Yes	N/A
[17]	Yes	No	46, 150	InSb–Au	99.9	9.6	N/A	Yes	N/A
[19]	Yes	No	90.4, 80	InSb-Teflon-Copper	94.0, 94.0	4.64, 8.36	N/A	Yes	0–45°
[33]	Yes	Yes	41.5, 150	InSb-Au-SiO_2_	98.95, 99.45	8.6, 12.8	1065.0, 499.0	Yes	0–60°
[34]	Yes	Yes	240, 120	InSb-InSb	99.9, 99.8	5.9, 6.4	1300.0, 1000.0	Yes	0–30°
[30]	Yes	Yes	15.8, 35	InSb-SiO_2_-Au	100	22	287	N/A	N/A
[35]	No	Yes	78, 150	InSb-Au	99.9	N/A	672.0	Yes	N/A
[36]	Yes	Yes	120, 400	SIS	N/A	0.46, 0.59	736.5, 661.3	N/A	N/A
[37]	Yes	No	195, 150	InSb	98.0, 92.0	10.12, 5.62	N/A	Yes	N/A
[38]	No	Yes	56.4, N/A	IATAI	94.20, 91.80	N/A	520.0, 810.0	N/A	N/A
This Work	Yes	Yes	43, 80	InSb–Copper	99.94	13.07	1146.0	Yes	0–45°

## Data Availability

The data presented in this paper were generated using the MATLAB 2023 computational tool and the CST electromagnetic simulation software 2024.

## Abbreviations

The following abbreviations are used in this manuscript:

MMA	Metamaterial Absorber
TMA	Terahertz Metamaterial Absorber
HMA	Hybrid Metamaterial Absorber
MSA	Metasurface Absorber
SRR	Square Ring Resonator
FWHM	Full Width Half Maximum
RIU	Refractive Index Unit
InSb	Indium Antimonide
Q-factor	Quality Factor
SIS	Semiconductor-Insulator-Semiconductor
IATAI	InSb1-Analyte1-(TiO2-Si)7-Analyte2-InSb2
